# Evaluation of a Virus-like Nanoparticle Porcine Circovirus Type-2 (PCV2) Capsid Protein Fused with the Pig Immunoglobulin Fc Fragment as a Novel Vaccine Candidate against PCV2 in Mice

**DOI:** 10.3390/vaccines9101128

**Published:** 2021-10-03

**Authors:** Qingping Luo, Waqas Ahmed, Yichen Dai, Ali Mohsin, Haifeng Hang, Yingping Zhuang, Meijin Guo

**Affiliations:** State Key Laboratory of Bioreactor Engineering, East China University of Science and Technology, Shanghai 200237, China; y10160102@mail.ecust.edu.cn (Q.L.); waqasahmed@mail.ecust.edu.cn (W.A.); y30181184@mail.ecust.edu.cn (Y.D.); alimohsin@ecust.edu.cn (A.M.); hanghaifeng@ecust.edu.cn (H.H.); ypzhuang@ecust.edu.cn (Y.Z.)

**Keywords:** PCV2, capsid (∆1-41aa)-pFc fusion protein, virus-like nanoparticles

## Abstract

Porcine circovirus Type 2 (PCV2) is a primary etiological pathogen of post-weaning multi-systemic wasting syndrome (PMWS). The capsid protein of PCV2 is the crucial immunogenic protein which can induce antibody generation and immune responses. However, there is still a lack of efficient PCV2 vaccines with high immunogenicity. In the current study, we developed a novel engineered PCV2 capsid (∆1-41aa)-pFc fusion protein (PCFP), which comprised a truncated capsid protein of PCV2 and a porcine IgG Fc fragment, fused to the capsid protein of PCV2 at the C-terminus. We found that this novel fusion protein could auto-assemble into virus-like nanoparticles with an estimated mean diameter of 22.6 nm, characterized by transmission electron microscopy. Immunization of BALB/c mice with this fusion protein significantly increased the production levels of anti-PCV2-capsid protein antibody in serum. Besides, the virus-like nanoparticles, PCFP was demonstrated to induce efficient cellular immune responses in mice, as evident by the high specific T cell reactivity to the PCFP fusion protein and the high production of the immune cytokines IFN-γ and IL-10 in an ex vivo re-stimulation system. Collectively, these findings demonstrate that the PCV2 truncated capsid subunit Fc-fusion protein can induce both cellular and humoral immune responses, and it displays great application potential.

## 1. Introduction

Porcine circovirus Type 2 (PCV2), a small non-enveloped single-stranded circular DNA virus, is considered to be a critical pathogen of several porcine circovirus-associated diseases (PCVAD) [1,2,3]. PCV2 has evolved into a series of new subtypes, such as PCV2a, PCV2b, PCV2c, PCV2d and PCV2e, since 1998, when it was identified. Currently, PCV2d has replaced PCV2b as the predominant PCV2 genotype in prevalence and circulation in swine herds [4,5,6,7,8]. 

Vaccination is generally considered as the most effective strategy for preventing PCV2 infection. However, the high mutation frequency of PCV2 creates great challenges in vaccine research and development [9]. In fact, a strain of PCV2b 41513 with new mutations has already been isolated from a failed case of a commercial PCV2 vaccine. The main mutation sites of this new strain were found to be largely limited to the capsid protein. Generally, the subtle changes in capsid genes lead to significant variations in their antigenicity or binding affinity to receptors, which finally results in immune escape [10]. To our knowledge, the currently available commercial PCV2 vaccines just target the PCV2a subtype. Therefore, it is necessary to explore novel PCV2 vaccines and further improve their immunogenicity to antagonize newly emerged strains.

Besides the inactivated PCV2 vaccine, PCV2 capsid protein has been the general target for vaccine development. PCV2 capsid protein is expressed in multiple protein expression systems including bacteria [11,12,13], yeasts [14,15], baculoviruses [16,17,18] and live-vectored expression systems [4,19], while it is very hard to produce the full-length PCV2 capsid protein in soluble form. It has been reported that immunization with a bacteria-expressed monomeric form of the PCV2 capsid protein could induce high titers of antibodies. However, the induced antibodies displayed low neutralizing activities. When produced in a baculovirus system, the vaccines exhibited better antibody induction effects. This evidence suggested that the host expression system would not only affect protein productivity but would also affect its immunogenicity, which would lead to variations in vaccine quality [20].

PCV2 capsid protein contains a major immunodominant epitope which is located between amino acids 65–87, 113–139 and 193–207, and is the only immunodominant structural protein of PCV2 [21]. Sequence analysis revealed that the region of the nuclear localization sequence (NLS) of PCV2 capsid protein is abundant in arginine residues and lacks certain codons for *E. coli*. Deletion of N-terminal arginine-rich NLS led to higher expression levels of the truncated capsid protein in *E. coli* and yeast host systems [15,22,23].

IgG Fc fusion protein can endow proteins with the IgG-like property of a long serum half-life. George et al. found that the fusion of G-CSF to human IgG domains resulted in homodimeric fusion proteins possessing high in vitro bioactivity, a long circulating half-life and enhanced hematopoietic properties in vivo [24]. In addition, Zhang et al. believed that the prolonged half-life of gp41Fc in the serum may enhance the generation of cross-reactive neutralizing antibodies [25]. Moreover, IgG Fc-mediated targeting Fcγ receptors (FcγR) on antigen-presenting cells (APCs) have already been demonstrated to enhance immune responses [26,27,28]. Therefore, we hypothesized that fusing PCV2 capsid protein with the IgG Fc fragment would be an effective strategy to enhance the efficiency of engineered vaccines because of the enhanced immune responses synergized by APC-mediated antigen presentation.

In the present study, we aimed to develop a novel PCV2 vaccine candidate with high immunogenicity. PCV2 capsid genes with N-terminal NLS sequence deletion were ligated with the Fc fragment of pig IgG1 to generate a fusion protein, namely PCV2 capsid (∆1-41aa)-pFc fusion protein (PCFP). This engineered fusion protein was successfully expressed in mammalian HEK293F cells. The purified fusion protein was observed to spontaneously self-assemble into virus-like nanoparticles similar to those of wild-type PCV2 virus. Compared with commercial subunit vaccines (CSV), the engineered fusion protein induced higher titers of PCV2-specific antibodies and intense cellular immune responses in immunized mice. These findings showed the promising potential of this novel PCV2 capsid (∆1-41aa)-pFc fusion protein as an efficient PCV2 vaccine candidate.

## 2. Materials and Methods

### 2.1. Vector Construction

The PCV2 genotype used in this study is from the virus strain of PCV2b 41513 (Genbank Accession No. ALD62452.1). The first 41 amino acids at the N-terminus of the PCV2b 41513 capsid protein were deleted and the truncated capsid gene of PCV2 was fused with the porcine IgG1 Fc fragment (GenBank Accession No. AAD38418.1) at the C-terminus by the GSGGGSGGGGSGGGS linker. A signal peptide with 19 amino acids (MGWSCIILFLVATATGVHS) was directly fused to the N-terminus of the truncated capsid gene of PCV2. The engineered fusion protein was named PCV2 capsid (∆1-41aa)-pFc fusion protein (PCFP). To construct the expression vector of the pEM-PCV2 capsid (∆1-41aa)-pFc fusion protein (pEM-PCFP), the recombinant PCFP genes were synthesized based on the bias codon for mammalian cells and then amplified by polymerase chain reaction (PCR) using a pair of oligonucleotide primers. The sequence of the forward primer was F: 5′-CTCACTATAGGGAGACCCAAGCTGGCTAGCCCGCCGCCACCATGGGCTGGAGCT-3′ and the reverse primer was R: 5′-ACTAGTGGATCCGAG-CTCGGTACCAAGCTTTTATCACTTGCCCTGGGTCTTGCT-3′. The amplified PCFP fusion genes were finally subcloned into the *Nhe*I/*Hin*dIII sites of pEM vector to obtain the recombinant pEM-PCFP vector.

The truncated capsid gene of PCV2 was also fused with murine IgG Fc (GenBank Accession No. CAD68963.1) at the C-terminus using the GSGGGSGGGGSGGGS linker. This engineered fusion protein was named PCV2 capsid (∆1-41aa)-mFc (PCFM) and applied in an in vivo immunogenicity evaluation in mice.

### 2.2. Cell Culture

Human embryonic kidney subclones, HEK293F (HEK293F, Thermo Fisher Scientific, Waltham, MA, USA), adapted to suspension growth in commercial serum-free medium, were used as host cells to express the PCFP fusion protein. HEK293F cells were cultured in Union-293 medium (Union-Biotech, Shanghai, China). Cell cultivation was performed in shaking flasks in an incubator shaker kept at 37 °C in a humidified atmosphere of 5% CO_2_. The shaking speed for the flasks was 100 rpm. Cell number and cell viability were determined by an automatic counting cell analyzer (model: CountStar Regel S3, Ruiyu, Shanghai, China).

### 2.3. Transfection Condition

To transiently transfect the HEK293F suspension cells, 40 kDa linear polyethylenimine (PEI) (Polysciences Inc., Warrington, PA, USA) was used as the transfection agent. Firstly, HEK293F cells were seeded at 1.0 × 10^6^ cells/mL and incubated overnight at 37 °C at an agitation speed of 110 rpm. The cells were then centrifuged at 100× *g* for 10 min and diluted at a density of 2.0 × 10^6^ cells/mL in fresh medium for use. Secondly, 1.0 μg of the pEM-PCFP expression plasmids were used per mL of the transfection volume. The pEM-PCFP plasmid was diluted with fresh medium to a concentration of 10 μg/mL. Next, PEI (PEI/DNA = 4:1, *w*/*w*) was added to the diluted DNA. The PEI/DNA complexes were mixed and incubated for 30 min and finally introduced to the resuspended HEK293F cell cultures.

### 2.4. Expression and Purification of Recombinant PCFP Fusion Protein in HEK293F Cells

After 96 h of post-transfection, the HEK293F cells were harvested by centrifugation (800× *g* for 10 min). PCFP fusion protein was purified with Protein A resin. Briefly, the cell pellets were resuspended in a cell lysis buffer containing 50 mM Tris, 300 mM NaCl, 1 mM PMSF and 1 % TritonX-100 (pH 8.0). After mixing, the suspended cells were lysed by ultrasonication, and the supernatant was harvested by centrifugation at 4 °C at 12,000 rpm for 20 min. Next, the supernatant was filtered through a 0.22 μm filter and loaded onto a Protein A column according to the Protein A affinity chromatography instructions. After washing the column with a wash buffer containing 20 mM Na_2_HPO_4_ and 150 mM NaCl (pH 7.0), the PCFP fusion protein was eluted with an elution buffer (100 mM glycine, pH 3.0) and neutralized with a neutralizer buffer (1 M Tris-HCl, pH 8.5). The protein fraction obtained was further dialyzed into a 1× PBS buffer containing 10% glycerol (pH 7.4) and filtered through a 0.22 μm membrane microfilter. The concentration of the purified fusion protein was quantified using a bicinchoninic acid assay kit (Beyotime, Shanghai, China).

### 2.5. SDS-PAGE and Western Blot Analysis

Both the transfected cell lysate and the enriched PCFP fusion protein fractions were analyzed by sodium dodecyl sulfate-polyacrylamide gel electrophoresis (SDS-PAGE) with 12.5% polyacrylamide gel (EpiZyme SCIENTIFIC, Shanghai, China). Proteins resolved on SDS-PAGE gel were transferred to a PVDF membrane (MILLLIPORE, Bedford, MA, USA) using the wet Western blot transfer mode. The transferred membrane was blocked with QuickBlock Western blocking buffer for 15 min at room temperature, followed by incubation with anti-porcine circovirus mAbs (1:1000 dilution; Abcam, Waltham, MA, USA) in TBST at 4 °C overnight. HRP-labeled goat anti-rabbit antibody (SAB, Shanghai, China) was used as the secondary antibody and was incubated with the membrane for 1 h at room temperature. After washing three times with TBST, protein bands were detected using an ECL chemiluminescence system (Beyotime, Shanghai, China) and the Tanon gel image system (GIS) software package (Tanon Company, Shanghai, China).

### 2.6. Morphology Analysis by Transmission Electron Microscope

Twenty microliters of the purified PCFP fusion protein or commercial vaccines were loaded onto a carbon-coated copper grid and incubated at room temperature for 15 min. The grids were then dried using filter paper, negatively stained with 3% phosphotungstic acid (PTA) for 10 min and observed using a transmission electron microscope JEM-1400 (JEOL, Tokyo, Japan) operating at 120 kV.

### 2.7. Immunization of Mice with Recombinant Fusion Protein

The 6–8-week-old female BALB/c and C57BL/6 mice were raised at the specific pathogen-free (SPF) grade animal laboratory. Thirty-five BALB/c mice were randomly divided into 7 groups (*n* = 5). The mice were injected intraperitoneally with 3 μg or 10 μg of PCFP fusion protein, or 10 μg of PCFM fusion protein. The commercial anti-PCV2 subunit vaccine (CSV, Erxing, Yebio Bioengineering Co., Ltd., Qingdao, China) at a dosage of 10 μg was applied as a positive control. PBS alone, PBS emulsified with Freund’s adjuvant (PBS + FA, also named the vehicle group) and 10 μg of pig IgG (pIgG) were used as negative controls. The recombinant proteins, including PCFP, PCFM and pig IgG, were diluted in 100 μL of PBS and then emulsified with 100 μL each of Freund’s complete adjuvant for the first immunization, and subsequently with 100 μL of Freund’s incomplete adjuvant as a booster at an interval of 12 days.

Fifteen C57BL/6 mice were randomly divided into 3 groups (*n* = 5). The mice were injected intraperitoneally with 10 µg of PCFM fusion protein with the same immunization strategy as that in BALB/c mice. PBS + FA was used as a negative control.

### 2.8. Evaluation of Specific Antibodies

The titer of PCV2d-capsid-specific IgG in serum collected from each mouse was measured using a commercial ELISA kit according to the manufacturer’s instructions (Meimian Biotechnology Co., Ltd., Yancheng, China). Briefly, microtiter plates were pre-coated with the PCV2 antigen. The mice serum was diluted 1:5 prior to incubation with the antigen. The HRP-anti-mouse IgG conjugate reagent was added to each well and incubated for 60 min at 37 °C. After washing, the chromogen solutions were added to each well for visualization. The optical density was measured using a microtiter plate reader (Thermo Fisher Scientific Inc., USA) at 450 nm.

### 2.9. Proliferation Assay and Cytokine Determination

Spleens of mice from each group were collected on Day 45 after the first immunization and homogenized in PBS. The resulting splenocytes were resuspended in RPMI 1640 medium supplemented with 10% *(v/v)* FBS, 100 U/mL penicillin and 100 μg/mL streptomycin, and plated into a 96-well U-bottomed plate at a density of 5 × 10^5^ cells per well. These splenocytes were then cultured at 37 °C in 5% CO_2_ in the presence of concanavalin A (ConA, 5 μg/mL), or the commercial anti-PCV2 subunit vaccine (CSV) or the fusion proteins, including PCFP and PCFM. Cell viability was measured after 72 h using Cell Titer Glo reagent according to the manufacturer’s instructions (Promega, G7572).

Cell culture supernatants were harvested after 72 h. Cytokine detection was performed using mouse interferon (IFN)-γ and interleukin (IL)-10 ELISA kits in accordance with the manufacturer’s protocol (Meimian Biotechnology Co., Ltd., Yancheng, China).

### 2.10. Flow Cytometry Analysis

Peripheral blood of mice was collected on Day 45 after first immunization. Erythrocytes in collected blood were lysed with a 1× RBC lysis buffer (Invitrogen, Cat#00-4333-57). After washing with cold PBS, the cells were incubated with PE-conjugated anti-CD3, APC-conjugated anti-CD4- and PE-Cy7-conjugated anti-CD8 antibodies (BD, Franklin Lakes, NJ, USA) at 4 °C for 30 min. The cells were then resuspended in a PBS buffer containing 1% FBS and analyzed with a BD Celesta flow cytometer.

### 2.11. Statistical Analysis

The distribution of the data was checked by the Shapiro–Wilk test. All the data passed the normality test. The statistical significance was analyzed using an unpaired two-tailed Student’s *t* test with Prism software (GraphPad). The statistical significance is shown in the figures and the figure legends. Error bars represent the standard deviation of the mean (SD). *p* values < 0.05 were considered statistically significant.

## 3. Results 

### 3.1. Construction of Recombinant Plasmid pEM-PCFP

**Figure 1:**
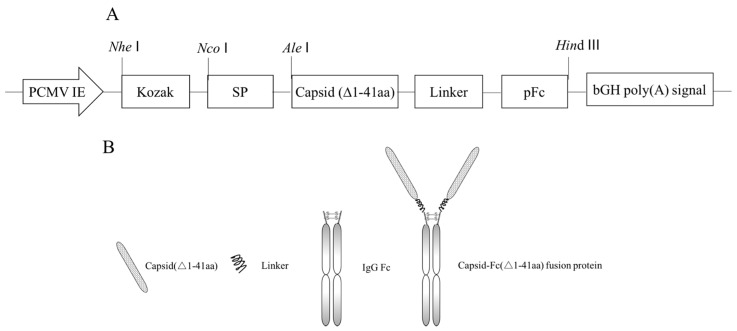
Schematic diagram of the expression vectors and fusion protein. (**A**) Gene expression cassette of the PCFP fusion protein. PCMV IE, the human cytomegalovirus immediately early promoter with the human cytomegalovirus immediate early enhancer region; Kozak, Kozak consensus; SP, Human IgG2 heavy chain signal peptide; Capsid (∆1-41aa), truncated (41 N-terminal amino acids) PCV2 capsid gene; Linker, linker peptides (GSGGGSGGGGSGGGS); pFc, Fc domain gene of the porcine IgG heavy chain. (**B**) Schematic representation of PCFP fusion protein. The PCFP fusion protein comprised a truncated PCV2 capsid and a porcine IgG Fc fragment which was fused to the capsid at the C-terminus via the linker (GSGGGSGGGGSGGGS).

The 41 amino acids at the N-terminus of the PCV2 capsid protein are a nuclear localization signal (NLS). To generate a novel PCV2 capsid fusion protein, the first 41 N-terminal amino acids of the PCV2 capsid were deleted and the truncated PCV2 capsid gene (GenBank Accession No. ALD62452.1) was fused with porcine IgG Fc at the C-terminus using the GSGGGSGGGGSGGGS linker. The resulting fusion protein was named PCFP. A schematic diagram of the gene expression cassette and fusion protein is illustrated in Figure 1.

The synthesized PCFP fusion gene was amplified by PCR (Figure S1A). The expected size of the PCR products was 1739 bp in length. The PCR product was subcloned into the restriction sites of *Nhe*I*/Hin*dIII in the pEM vector. The recombinant plasmid was characterized by double digestion with *Nhe*I*/Hin*dIII restriction enzymes, resulting in a band of approximately 1739 base pairs in length, as expected (Figure S1B). In addition, the recombinant plasmid was further verified by DNA sequencing (data not shown).

### 3.2. Production and Purification of PCFP Fusion Protein

Full-length PCV2 capsid protein has been found to be expressed in soluble form at quite low levels in various expression systems such as Pichia pastoris yeast, baculoviruses, Sf9 insect cells, *E. coli*, etc. To mitigate this issue, the 41 N-terminal amino acids of PCV2 capsid protein for nuclear localization signal were deleted and mammalian HEK293F cells were applied as the expression system. Transient expression of PCFP fusion protein in the HEK293F cells was analyzed by SDS-PAGE and Western blot analyses. As shown in Figure S2, a protein band with a molecular weight of 59 kDa was observed, especially in the elution fractions from the Protein A resin column, indicating successful expression and purification of the PCFP fusion protein in the HEK293F cells. The product was further concentrated by dialysis and quantified by a BCA protein assay for the following in vitro and in vivo evaluation. 

### 3.3. PCFP Fusion Protein Spontaneously Assembled into Virus-Like Nanoparticles

**Figure 2:**
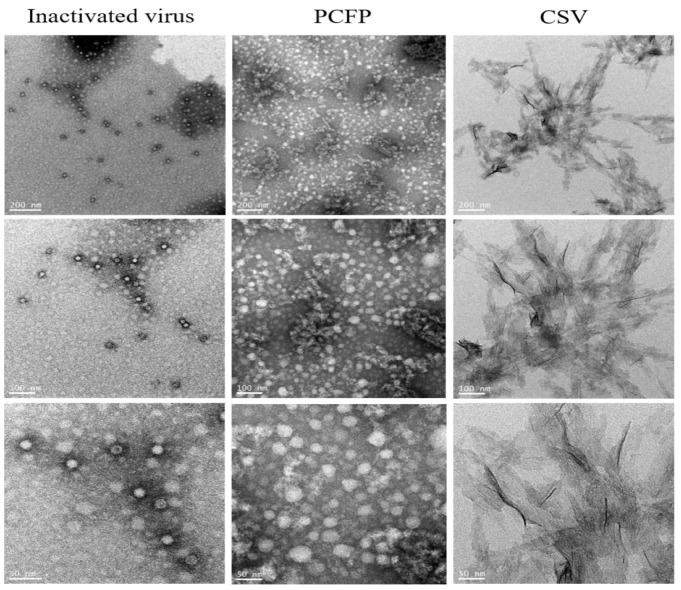
PCFP fusion protein spontaneously assembled into virus-like nanoparticles. The morphology of the purified PCFP protein was analyzed using a transmission electron microscope, with the inactivated virus and a commercial subunit capsid protein as reference controls. Morphological analysis indicated that PCFP fusion protein formed virus-like nanoparticles with a similar morphology to that of the original virus.

To determine whether recombinant PCFP fusion protein from a mammalian cell expression system could form virus-like nanoparticles, we characterized the purified PCFP fusion protein using transmission electron microscopy with inactivated PCV2 virions and a commercial vaccine (PCV2 subunit capsid protein, CSV) as positive controls. As shown in Figure 2, the PCFP fusion protein formed virus-like nanoparticles with an estimated mean diameter of 22.6 nm and a similar morphology to that of inactivated PCV2 virions with a diameter of 17 nm. However, the commercial vaccine did not show the typical morphology.

### 3.4. PCFP Induced Neutralizing Antibodies in Immunized Mice

**Figure 3:**
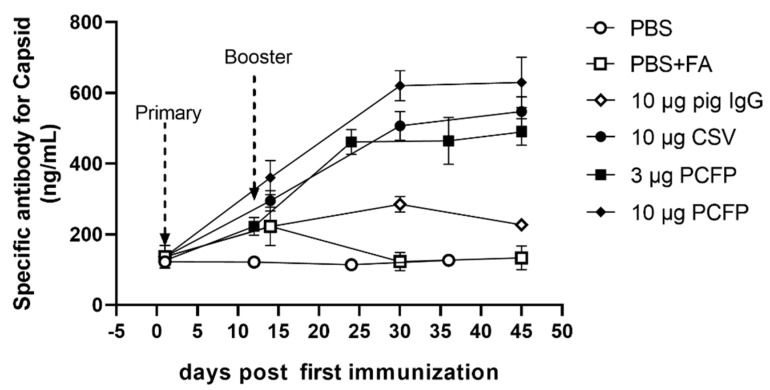
Kinetics of the specific antibody titer for the PCV2 capsid in the serum of BALB/c mice immunized with PCFP. The titer of PCV2b−capsid−specific IgG in serum from immunized mice was measured by ELISA.

Sera from mice immunized with the PCFP fusion protein or the commercial PCV2 vaccine (CSV) were subjected to an ELISA test to evaluate specific antibody production against the PCV2 capsid. In this ELISA assay, the recombinant PCV2 capsid protein without the Fc tag was used as the coating antigen to eliminate interference from Fc fragments. As shown in Figure 3, significantly higher titers of anti-PCV2 antibodies were produced in the BALB/c mice immunized with the PCFP recombinant fusion protein and CSV compared with the control mice. The IgG antibody titers in the mice immunized with PCFP and CSV exhibited a similar trend, which increased rapidly from 14 days after the first immunization (dpi) and reached the highest level by 45 dpi. Noticeably, PCFP induced specific IgG antibody production in a dose-dependent manner, the antibody concentrations being 620.2 ng/mL and 464.4 ng/mL for 10 μg PCFP and 3 μg PCFP, respectively.

To further evaluate the effects of the Fc fragment on the fusion protein-mediated immunogenicity, we engineered the PCFP fusion protein with murine IgG Fc instead of pig IgG Fc. The engineered fusion protein was denoted PCFM (PCV2 capsid (∆1-41aa)-Fc (murine)). As shown in Figure S3, higher levels of IgG antibody titers in immunized BALB/c mice from the PCFM group were observed as early as 14 dpi than in those from the PCFP group and maintained high levels at the last detection timepoint (45 dpi). Meanwhile, similar kinetics of the specific IgG antibody titers were observed in C57BL/6 mice immunized with PCFM. These results indicated the potential contribution of the homologous IgG Fc fragment in inducing robust humoral immune responses.

### 3.5. PCFP Changed Immuno-Profiling of Peripheral T Lymphocytes in Immunized Mice

**Figure 4:**
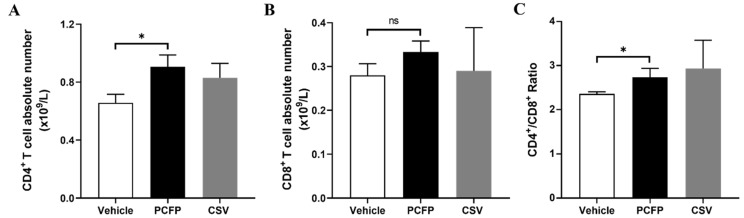
FACS analysis of T lymphocytes in the peripheral blood of immunized mice. (**A**,**B**) Absolute numbers of peripheral CD4+ T and CD8+ T cells were analyzed by flow cytometry and cell counting. (**C**) The ratio of CD4+ T cells to CD8+ T cells was analyzed. The data are shown as means ± SD and were analyzed using Student’s t-test. * *p* < 0.05 was considered statistically significant. ns, no statistical difference.

Peripheral blood samples from the mice immunized with PCFP fusion protein or CSV were analyzed for the absolute numbers of the CD4^+^ T and CD8^+^ T cell populations with flow cytometry. The results showed that the absolute number of CD4^+^ T cells in the PCFP group was significantly higher than that in the vehicle control group (also named the PBS + FA group) (*p* < 0.05, Figure 4). Although no significant differences were observed in the absolute number of CD8^+^ T cells among the groups, the ratio of CD4^+^/CD8^+^ T cells was also significantly higher in the PCFP group than in the vehicle control group (*p* < 0.05), suggesting that immunization with PCFP fusion protein changed the peripheral T lymphocyte profile. 

### 3.6. PCFP Immunization Elicited Specific Lymphocyte Proliferative Responses

**Figure 5:**
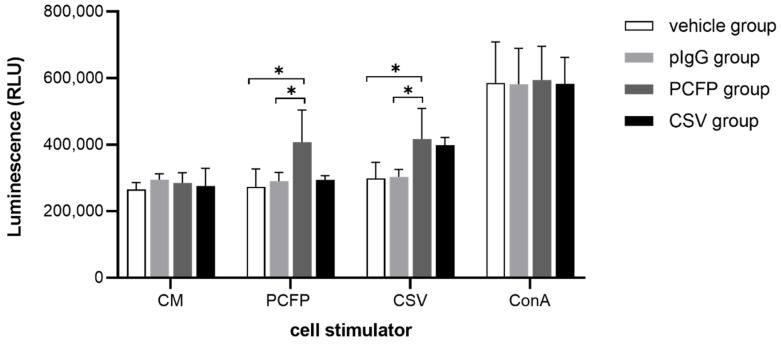
PCFP immunization elicited a specific lymphocyte proliferative response in an ex vivo splenocyte stimulation setting. Splenocytes from immunized mice were stimulated in vitro with the subunit vaccine CSV, PCFP fusion protein or ConA. Cell viability was measured after 72 h using Cell Titer Glo reagent. The data are shown as the mean ± SD, and were analyzed using Student’s t-test for comparisons between two groups. * *p* values < 0.05 were considered statistically significant.

To analyze PCFP immunization-induced specific T-cell responses against PCV2 capsids, splenocytes from immunized mice were stimulated in vitro with the subunit vaccine CSV, PCFP fusion protein or ConA. As expected, we found that the positive proliferative responses were comparable among all the tested groups following ConA stimulation. There was a significant increase in splenocyte proliferation in the PCFP group with PCFP fusion protein or CSV stimulation compared with the vehicle or pig IgG groups. Moreover, the proliferative responses were comparable in the PCFP group and the CSV group in response to CSV stimulation (Figure 5). These results demonstrated that PCFP immunization could effectively induce specific cellular immune responses.

### 3.7. Cytokine Evaluation

**Figure 6:**
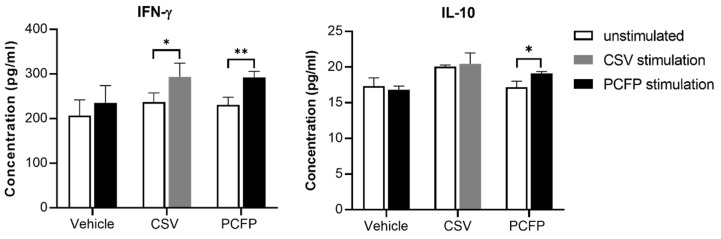
ELISA analysis of IFN-γ and IL-10 cytokines in the supernatant of ex vivo splenocyte cultures. Splenocytes isolated from immunized mice were cultured in the presence of CSV or PCFP for 72 h. The cytokine levels of IFN-γ and IL-10 was determined by ELISA. The data are shown as the mean ± SD, and were analyzed using Student’s t-test. * *p* < 0.05, ** *p* < 0.01.

To evaluate cytokine responses against PCFP immunization, we assessed IFN-γ and IL-10 levels in the culture supernatant of splenocytes stimulated with PCV2 antigen in vitro. As shown in Figure 6, the IFN-γ levels produced by lymphocytes from the PCFP and CSV groups significantly increased following either PCFP or CSV stimulation compared with those from the vehicle group. Significant increases were also observed for IL-10 levels in the lymphocyte cultures from the PCFP group in response to PCFP stimulation (Figure 6). However, IL-10 production by lymphocytes from the CSV group had no significant changes in response to CSV stimulation as compared with the base levels. The results indicated that immunization with PCFP can enhance Th1/Th2 responses.

## 4. Discussion

Porcine circovirus 2 (PCV2) can lead to PCVAD and has a severe impact on the swine industry [29]. Today, vaccination is a major prophylactic strategy for PCVAD disease control. A variety of PCV2 vaccines have been developed, such as inactivated vaccines, subunit vaccines and nucleic acid vaccines, etc. [30,31,32,33]. Currently, commercial PCV2 subunit vaccines have been developed on the basis of the PCV2a strain. However, PCV2 has been evolving into new subtypes from PCV2a, resulting in the failure of current PCV2 vaccines.

Given that PCV2b was once the predominant subtype and had been isolated from the failed case of commercial PCV2 vaccines, here, we explored a novel PCV2 vaccine candidate based on the capsid protein sequence of a PCV2b 41513 strain obtained from a vaccine failure case. The candidate was designed as a fusion protein comprising a N-terminal truncated capsid protein and a porcine IgG1 Fc fragment, which was fused to the capsid protein at the C-terminus. The NLS sequence at the N-terminus of the PCV2b capsid protein provided nuclear localization signals. Therefore, deletion of this sequence would enhance the expression levels of the engineered fusion protein [22]. It is well known that antigen-presenting cells (APCs) express high levels of Fc-gamma receptors. When engaging with Fc-tagged PCV2 protein, APCs could synergize with T cells to induce intense anti-PCV2 specific immune responses.

The engineered PCFP fusion protein was successfully expressed in mammalian HEK293F cells. To our knowledge, this was the first time that PCV2 protein has been expressed in mammalian cells. The target protein was expressed in both intracellular and extracellular forms and spontaneously assembled into virus-like nanoparticles. The in vivo studies showed that immunization with PCFP fusion protein could effectively induce high titers of PCV2 capsid-specific antibodies in the immunized mice. Compared with the commercial subunit vaccine (CSV), PCFP fusion protein immunization elicited specific T lymphocyte proliferative responses in immunized mice, as shown in the ex vivo re-stimulation assay. Consistently, significant increases in the absolute cell number of CD4^+^ T cells as well as the CD4^+^/CD8^+^ ratio were observed in the peripheral blood of immunized mice, as analyzed by flow cytometry. Furthermore, immunization with the PCFP fusion protein significantly enhanced the secretion of IFN-γ, a pro-inflammatory cytokine, which is generally produced by Th1 cells, indicating that the major subpopulation (Th1 cells) of T helper cells was activated by the exogenous fusion protein. In conclusion, the new PCFP fusion protein developed here can effectively trigger cellular and humoral immune responses in mice. It is worthy to note that in this “proof of concept” study, we verified the feasibility of this novel design for a PCV2 vaccine candidate. Our confirmation of the immunogenicity and immune-provoking properties of the vaccine candidate may prompt us to further clarify its protective efficacy in the future. IL-10 is an immune regulatory cytokine associated with Th2 immune responses. Unexpectedly, a significant difference in the secretion of cytokine IL-10 was also observed in re-stimulated splenocytes from mice immunized with the PCFP fusion protein, whereas in the case of the CSV group, there were no changes in the IL-10 levels. The higher levels of IL-10 induced by the PCFP fusion protein may be attributed to the extra biological activity of the unique IgG1 Fc fragment in this fusion protein.

PCV2 capsid is composed of 60 capsid protein subunits with icosahedral symmetry. The N-terminal region of the PCV2 capsid protein, which is rich in basic amino acids, can bind PCV2 genomic DNA and facilitate virus packaging [3,34]. Recently, researchers have identified the structural roles of the N-terminal region of PCV2 capsid protein in assembly. When the N-terminal region of PCV2 capsid protein was truncated by more than 30 residues, virus-like nanoparticles were not formed. It was speculated that the stable virus-like nanoparticles of the PCV2 capsid protein depend on the interaction between 15PRSHLGQILRRRP27 (α-helix) and 33RHRYRWRRKN42 (NLS-B) in a repeated manner [35]. In this study, PCFP fusion protein was constructed by ligation of a truncated PCV2 capsid protein with a porcine IgG1 Fc fragment. It self-assembled into virus-like nanoparticles with a morphology similar to the naïve PCV2 virus. The results in Figure 2 strongly suggest that IgG1 Fc fragments could assist the PCFP fusion protein in self-assembly and the formation of stable virus-like nanoparticles.

Several studies have demonstrated that an antigen’s conformational stability is important for APC-mediated antigen processing because it is closely related to immunogenicity and immune polarization [36,37,38]. As we know, empty capsids are physically unstable compared with virus particles. In this study, fusion with a Fc fragment led to the formation of multiple disulfide bridges, which resulted in greater stability of the capsid protein. In addition, Fc-gamma receptors (FcγRs) are expressed on dendritic cells (DCs), which are professional antigen-presenting cells (APCs) and play an important role in priming the T cell response against pathogens and tumors. Many studies have demonstrated that immunogens engaging with FcγR on APCs can be highly specifically captured and presented, leading to enhanced cellular immune responses [27,39,40]. Therefore, it could be speculated that the Fc fragment in the PCFP fusion protein involves APCs, further enhancing the immune responses against the target pathogens.

## 5. Conclusions

Taken together, the engineered PCFP fusion protein could spontaneously form virus-like nanoparticles. These virus-like nanoparticles were demonstrated to induce not only the production of specific-PCV2 capsid antibodies but also enhanced Th1/Th2 responses, indicating that the PCFP fusion protein can elicit humoral and cellular immune responses. Further studies, including a planned pig challenge study, will allow full validation of PCFP-mediated protective activity against PCV2. In conclusion, our results suggest that the PCFP fusion protein has development potential and may be a valuable model for exploring more novel PCV2 subunit vaccine candidates.

## Data Availability

The data presented in this study are available on request from the corresponding author. The data are not publicly available due to intellectual property considerations.

## Supplementary Materials

The following are available online at https://www.mdpi.com/article/10.3390/vaccines9101128/s1, Figure S1: Construction of the recombinant plasmid pEM-PCFP, Figure S2: Purification of PCFP fusion protein expressed in HEK 293F cells by a Protein A affinity chromatography column, Figure S3: Kinetics of the specific antibody titer for PCV2 capsid in the serum from mice immunized with PCFM.
