# Supplementary material for: Evaluation of a Virus-like Nanoparticle Porcine Circovirus Type-2 (PCV2) Capsid Protein Fused with the Pig Immunoglobulin Fc Fragment as a Novel Vaccine Candidate against PCV2 in Mice
Source: Vaccines (Basel). 2021 Oct 3;9(10):1128. doi: 10.3390/vaccines9101128 (PMC8540293; doi:10.3390/vaccines9101128)
Supplement: Supplementary file 1 [file vaccines-09-01128-s001.zip › vaccines-1307946-supplementary.pdf]

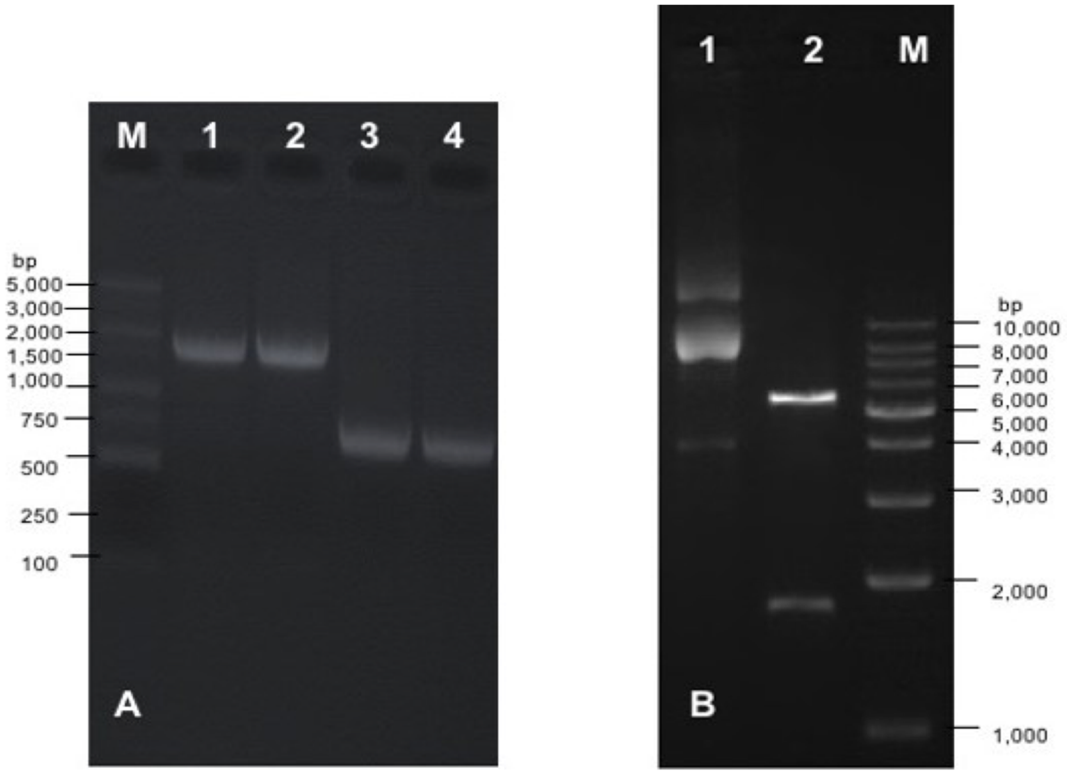

Figure S1. Construction of the recombinant plasmid pEM-PCFP. DNA electrophoresis analysis of PCFP gene PCR product and the pEM-PCFP plasmid. (A) PCV2 Capsid (△1-41aa)-pFc gene was confirmed by PCR. M, DNA marker (1kb DNA ladder); Lane 1-2, PCFP gene PCR product; Lane 3-4, truncated PCV2 capsid gene PCR product. (B) The recombinant plasmids were extracted and digested with *Nhe* I/*Hind*III enzymes. M, DNA Marker 5000; Lane 1, full-length of pEM-PCFP plasmids; Lane 2, The recombinant plasmids pEM-PCFP were cleaved by restriction enzymes *Nhe*I and *Hin*dIII.

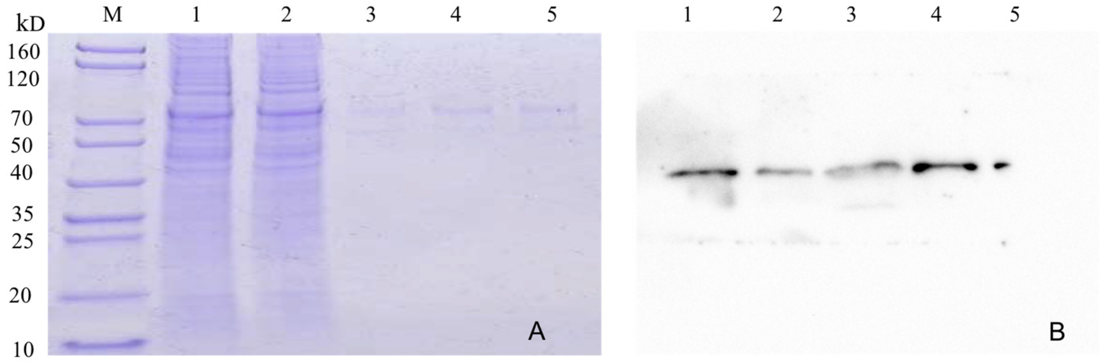

Figure S2. Purification of PCFP fusion protein expressed in HEK 293F cells by a Protein A affinity chromatography column. Cell lysates of HEK293F cells transfected with the recombinant plasmids and purified PCFP proteins were run on a 12.5% polyacrylamide gel under denaturing conditions. Gel was stained with Coomassie blue for SDS-PAGE analysis (A) and transferred to a nitrocellulose membrane for western blot analysis using anti-capsid antibody (B). M, protein marker; Lane 1 to 5, cell lysates, flow through, elution fraction 1, elution fraction 2, elution fraction 3, respectively.

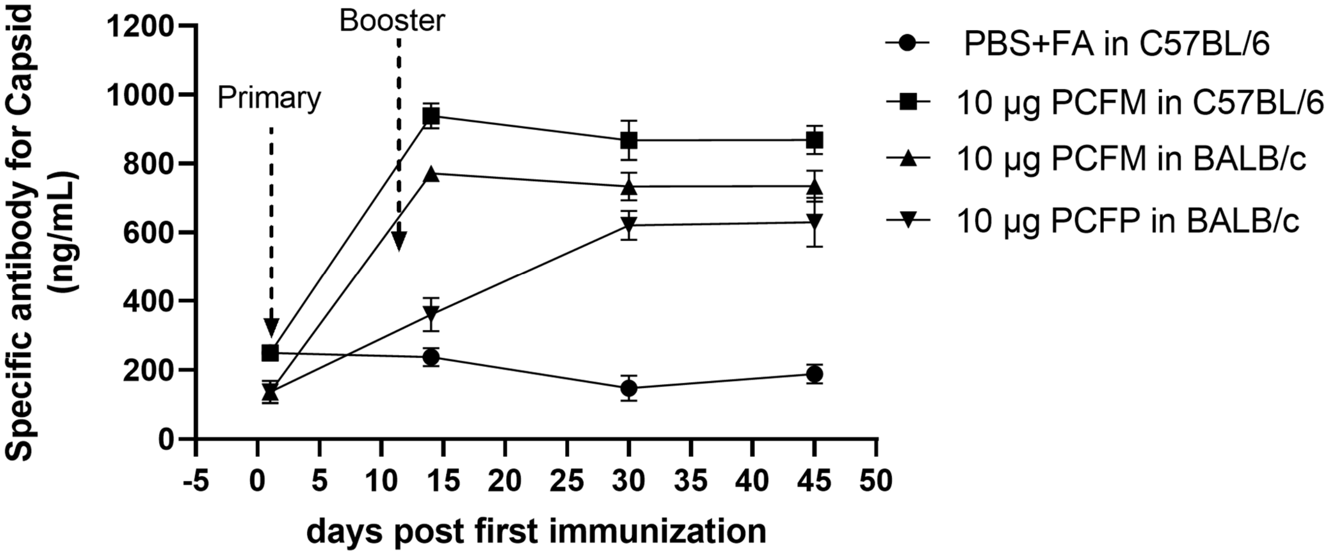

Figure S3. Kinetics of the specific antibody titer for PCV2 capsid in the serum from mice immunized with PCFM. The titer of PCV2b-capsid-specific IgG in serum from immunized mice was measured by ELISA.
